# Anti-Biofilm Activity of Cannabigerol against *Streptococcus mutans*

**DOI:** 10.3390/microorganisms9102031

**Published:** 2021-09-25

**Authors:** Muna Aqawi, Ronit Vogt Sionov, Ruth Gallily, Michael Friedman, Doron Steinberg

**Affiliations:** 1 The Biofilm Research Laboratory, The Faculty of Dental Medicine, The Institute of Dental Sciences, The Hebrew University of Jerusalem, Jerusalem 9112102, Israel; ronitsionov@gmail.com (R.V.S.); dorons@ekmd.huji.ac.il (D.S.); 2 The Institute of Drug Research, School of Pharmacy, Faculty of Medicine, The Hebrew University of Jerusalem, Jerusalem 9112102, Israel; michaelf@ekmd.huji.ac.il; 3 The Lautenberg Center for General and Tumor Immunology, The Hadassah Medical School, The Hebrew University of Jerusalem, Jerusalem 9112102, Israel; ruthg@ekmd.huji.ac.il

**Keywords:** biofilm, Cannabigerol, dental caries, *Streptococcus mutans*

## Abstract

*Streptococcus mutans* is a common cariogenic bacterium in the oral cavity involved in plaque formation. Previous studies showed that Cannabigerol (CBG) has bacteriostatic and bacteriocidic activity against *S. mutans*. The aim of the present study was to study its effect on *S. mutans* biofilm formation and dispersion. *S. mutans* was cultivated in the presence of CBG, and the resulting biofilms were examined by CV staining, MTT assay, qPCR, biofilm tracer, optical profilometry, and SEM. Gene expression was determined by real-time qPCR, extracellular polysaccharide (EPS) production was determined by Congo Red, and reactive oxygen species (ROS) were determined using DCFH-DA. CBG prevented the biofilm formation of *S. mutans* shown by reduced biofilm biomass, decreased biofilm thickness, less EPS production, reduced DNA content, diminished metabolic activity, and increased ROS levels. CBG altered the biofilm roughness profile, resulting in a smoother biofilm surface. When treating preformed biofilms, CBG reduced the metabolic activity of *S. mutans* with a transient effect on the biomass. CBG reduced the expression of various genes involved in essential metabolic pathways related to the cariogenic properties of *S. mutans* biofilms. Our data show that CBG has anti-biofilm activities against *S. mutans* and might be a potential drug for preventive treatment of dental caries.

## 1. Introduction

A biofilm is an architectural colony of microorganisms wrapped in a matrix of extracellular polymeric substances that are produced by them [1]. The biofilm protects the bacteria from environmental stress stimuli. Sessile cells embedded in the biofilm are up to 1000 times more resistant to antibiotics than cells in their planktonic state [2]. These structures also enable communication between the microorganisms in a process termed quorum sensing (QS) [3].

The oral microbiota grows on surfaces as structurally and functionally organized communities of interacting species in biofilms termed dental plaque [4]. Dental biofilms can range from 50 cell layers in thinner plaques to more than 100 cell layers in thicker biofilms [5]. They can form on many different surfaces in the mouth: enamel, artificial dentures, orthodontic devices, or implants [6,7,8]. Oral diseases such as tooth decay, gingivitis, candidiasis, and periodontal diseases are associated with the presence of different and specific types of microorganisms [9]. The formation of biofilms on tooth surfaces is a predominant factor in the etiology of dental caries [10] and periodontal diseases [11].

The oral cavity is unique due to the diversity of surfaces found in the mouth. For example, the use of fixed orthodontic appliances to the oral cavity not only promotes the amount of biofilm formation but also increases the level of acidity, resulting in a higher cariogenic challenge around the appliances [12]. The material and surface properties of orthodontic brackets/resin can influence bacterial attachment, plaque retaining capacity, and microbial diversity [13,14]. Moreover, bacterial adhesion forces to composite resin, often having a rougher surface than enamel or brackets, may be stronger than those to brackets or saliva-coated enamel [15].

*Streptococcus mutans* (*S*. *mutans*) is considered the most important etiological microbe of dental caries [16]. *S. mutans* is a Gram-positive, facultative anaerobic bacterium with acidogenic and aciduric properties [17,18]. *S. mutans* is a key contributor to the formation of the pathogenic dental biofilms, mainly due to its ability to synthesize extracellular polysaccharides [19] such as water insoluble glucans or fructans by the action of glucosyltransferases (GTFs) and fructosyltransferase (FTF). *S. mutans* also produces multiple glucan-binding proteins (Gbp proteins), which are thought to promote adhesion to matrix glucans and to shape the overall architecture of the biofilm [20,21]. Moreover, it produces lactic acid through the metabolism of carbohydrates, leading to the development of tooth decay [22].

Saliva plays an important role in *S. mutans* biofilm formation. Oral streptococci interact with the enamel salivary pellicle to form biofilm on tooth surfaces. Streptococcal cell wall components such as adhesins mediate adherence to various salivary molecules, especially sugars or oligosaccharides [23]. *S. mutans* colonization in NOD/SCID.*e2f1*^−/−^ mice that show hyposalivation was significantly increased when mice were pre-treated with human saliva or commercial salivary components [24]. Following initial adherence, bacteria grow and survive only if the physical and chemical environment such as pH, oxygen levels, and redox potential is conducive [23]. An interaction between the salivary parameters such as calcium, inorganic salivary phosphate, salivary pH, and the dental caries has been established. The mean values of the salivary parameters were statistically higher in cario-resistant subjects in comparison to cario-active subjects [25].

Recently, patients and physicians are turning towards the use of natural extracts, such as herbal products, for prophylaxis and treatment of different diseases [26]. The *Cannabis sativa* plant has been used for thousands of years for recreational and therapeutics purposes as well many other uses [27]. The *Cannabis* plant contains more than 120 terpenophenolic constituents named phytocannabinoids. Among the many available cannabinoids, Cannabigerol (CBG), a non-psychotropic Cannabis-derived cannabinoid, has received much attention.

In vitro and in vivo studies suggest a potential future for CBG to address unmet needs in medical therapy. Accumulating data indicate that CBG may have therapeutic potential in treating neurological disorders (e.g., Huntington’s disease, Parkinson’s disease, and multiple sclerosis) and inflammatory bowel disease [28]. To date, only a few studies have investigated its anti-bacterial activity. Appendino et al. [29] demonstrated an anti-bacterial activity of CBG against methicillin-resistant *Staphylococcus aureus* (MRSA). Using a systemic *S. aureus* infection model in mice, Farha et al. [30] showed that CBG was as effective at reducing colony forming units as vancomycin. Recently, two studies have tested the potential use of CBG as an anti-bacterial agent against dental plaque-associated bacteria [31,32]. Vasudevan et al. [32] found that CBG infused mouthwash products showed similar bactericidal efficacy as that of chlorhexidine 0.2%, while Stahl et al. [31] found that cannabinoids were more effective in reducing the bacterial colony count in dental plaques compared with the well-established synthetic oral care products such as Oral B and Colgate. We have demonstrated that CBG has an anti-quorum sensing effect against *Vibrio harveyi* [33]. Furthermore, we have shown that CBG exerts anti-bacterial effects towards planktonic *S. mutans* [34]. Here, we tested the potential use of CBG as an anti-biofilm agent against *S. mutans* biofilms as a mean to combat dental plaque.

## 2. Materials and Methods

### 2.1. Materials

Cannabigerol (CBG) (hemp isolate, 95% purity) was purchased from NC Labs (Prague, Czech Republic) and dissolved in ethanol at a concentration of 10 mg/mL. Respective dilutions of ethanol were used as control. 3-(4,5-Dimethyl-2-thiazolyl)-2,5-diphenyl-2H-tetrazolium bromide (MTT) and 20,70-dichlorofluorescein diacetate (DCFH-DA) were obtained from Sigma-Aldrich, St. Louis, MO, USA.

### 2.2. Bacterial Growth and Biofilm Formation

*S. mutansUA159* were grown overnight at 37 °C in 95% air/5% CO_2_ in brain heart infusion broth (BHI, Acumedia, Lansing, MI, USA) until an OD_600nm_ of approximately 1 was reached [35]. For biofilm formation, the overnight culture was diluted 1:10 in BHI containing 2% sucrose. Two hundred microliters of the bacterial culture were seeded in each well of a flat-bottom 96-well microplate (Corning, Glendale, AZ, USA) in the absence or presence of increasing concentrations of CBG (1–5 μg/mL) or respective ethanol concentrations and incubated at 37 °C in 95% air/5% CO_2_ for 24 h. At the end of incubation, the medium was removed, and the biofilms formed at the bottom of the wells were washed twice with 200 µL phosphate-buffered saline (PBS) to remove any remaining planktonic bacteria. Untreated and ethanol-treated bacteria served as controls.

### 2.3. Crystal Violet (CV) Staining of Biofilms

The biofilms were stained with 200 µL of 0.1% Crystal Violet (CV) that was prepared from a 0.4% Gram’s crystal violet solution (Merck, EMD Millipore Corporation, Billerica, MA, USA) diluted with DDW [36]. After a 15 min incubation at room temperature, the CV was removed, and the wells were washed twice with DDW and dried overnight. Extraction of the CV stain was performed by adding 150 µL of 33% acetic acid to the wells followed by a 5 min incubation under constant shaking. The absorbance was measured at 595 nm using the M200 Tecan plate reader (Tecan Trading AG, Männedorf, Switzerland), which provides a measure of the amount of biofilm biomass.

### 2.4. DNA Quantification in Biofilms

Biofilms that have been formed in 48-well tissue culture plates (Corning) following the incubation of 500 μL *S. mutans* in BHI with 2% sucrose in the absence or presence of CBG or respective ethanol concentrations were washed twice with 500 μL PBS followed by lysis in 200 μL of 0.04M NaOH for 1 h at 60 °C in a water bath under constant shaking. At the end of incubation, 18.5 µL of 1M Tris-HCl pH 7 were added to neutralize the NaOH [37]. The amount of DNA in each sample was quantified by qPCR with specific primers for *S. mutans* 16S rRNA (Table 1) using Power Green Master Mix (Applied Biosystems, Waltham, MA, USA). The PCR amplification was performed in a Bio-Rad CFX Connect Real-time system with the Bio-Rad CFX Maestro program. The amount of DNA was quantified according to the standard curve obtained using known DNA concentrations of purified *S. mutans* DNA. Purified DNA was extracted from an overnight culture of *S. mutans* using GenElute Bacterial Genomic DNA kit (Sigma Aldrich, St. Louis, MO, USA) as per the manufacturer’s instructions.

### 2.5. Tetrazolium Reduction Assay (MTT Metabolic Assay)

This colorimetric assay is useful for measuring the metabolic activity of bacteria. The MTT metabolic assay was performed as previously described [38]. Briefly, 50 µL of a 0.5 mg/mL MTT (Calbiochem, Darmstadt, Germany) solution in PBS was added to the biofilms in 96-well plates. After 1 h incubation at 37 °C, the wells were washed with PBS, and the tetrazolium precipitates contained in the biofilms were extracted with 150 µL dimethylsulfoxide (DMSO) (Bio-Lab Ltd., Jerusalem, Israel). After 10 min on an orbital shaker, the absorbance was measured at 570 nm using the M200 Tecan plate reader.

### 2.6. FilmTracer SYPRO Ruby Biofilm Matrix Stain

SYPRO Ruby stain labels most classes of proteins and is used to stain proteins in the extracellular matrices of bacterial biofilms. The washed biofilms were stained with 100 μL of the FilmTracer SYPRO Ruby biofilm matrix stain (Invitrogen, Molecular Probes, Eugene, OR, USA) for 30 min at room temperature followed by several washes with DDW [39]. The fluorescence intensity of the stained biofilms was measured in the M200 Tecan microplate reader with an excitation at 450 nm and an emission at 610 nm.

### 2.7. High-Resolution Scanning Electron Microscopy (HR-SEM)

Biofilms were allowed to form on sterile circular glass pieces in the absence or presence of various concentrations of CBG. After a 24 h incubation, the glass specimens were rinsed with DDW and fixed in 4% glutaraldehyde in DDW for 40 min. The glass specimens were washed again with DDW and allowed to dry at room temperature. The specimens were then mounted on a metal stub and sputter coated with iridium and visualized by a high-resolution scanning electron microscope (Magellan XHR 400L, FEI Company, Hillsboro, OR) [34]. Three specimens from each treatment group were prepared and examined under SEM to evaluate the effect of CBG on biofilm formation.

### 2.8. RNA Extraction

RNA extraction was performed as previously described [33] with slight modifications. An overnight culture of *S. mutans* (OD_600nm_ ~ 1) was diluted 1:10 in BHI containing 2% sucrose and the biofilms were allowed to form in the absence or presence of CBG (2.5 μg/mL) or respective ethanol concentrations (0.025%) by incubating the bacteria at 37 °C in 95% air/5% CO_2_ for 24 h. At the end of incubation, the biofilms were washed twice with PBS and soaked in 2 mL of RNA Protect (Qiagen, Hilden, Germany) for 5 min at room temperature, and the RNA isolation was performed using the RNeasy MINI kit (Qiagen) including on-column DNase digestion according to the manufacturer’s instructions. RNA purity and quantity were determined using Nanodrop (Nanovue, GE Healthcare Life Sciences, Buckinghamshire, UK). Only samples with an OD_260nm_/OD_280nm_ ratio of 2 and an OD_260nm_/OD_230nm_ ratio above 1.8 were used for cDNA synthesis.

### 2.9. Reverse Transcription (RT) and Quantitative Real-Time PCR

The RNA was converted to cDNA using the cDNA qScript cDNA synthesis kit (QuantaBio, Beverly, MA, USA). The relative expression levels of target genes were analyzed by Bio-Rad CFX Connect Real-time system with the Bio-Rad CFX Maestro program. Power SYBR Green PCR Master mix (Applied Biosystems, Waltham, MA, USA) was used to amplify the genes of 10 ng cDNA per well in combination with 300 nM of respective F/R primer set (Table 1). The PCR cycle involved initial heating at 50 °C for 2 min and an activation step at 95 °C for 10 min, followed by 40 cycles of amplification (95 °C for 15 s and 60 °C for 1 min), and the dissociation curve was determined by initial heating at 95 °C for 15 s, followed by 10 s at 60 °C, and 0.5 temperature increments until 95 °C was reached. The 16S rRNA expression was used for normalization and to calculate the relative changes in target gene expression using the 2^−ΔΔCt^ method. Control reactions were performed with RNA that had not been reverse-transcribed to ensure that no genomic DNA was amplified during the PCR process. Gene expression was expressed in relative values, setting the expression level of the untreated and ethanol-treated control to 1 for each gene. The assays were performed in triplicates and repeated three times [40].

### 2.10. Optical Profilometry

The *S. mutans* biofilms were prepared as described above in the presence of different concentrations of CBG or ethanol on sterile glass pieces. Following a 24 h incubation, the biofilms were washed twice with PBS before drying them with liquid nitrogen. The biofilms were then visualized under an optical profilometer (Optical Profilometer Contour GT-K1, Bruker Corporation, Billerica, MA, USA). The images were later processed using Vision 64 program. The F-operator and the Gaussian Regression filters were applied on the images to calculate the surface roughness.

### 2.11. Determination of Extracellular Polysaccharide (EPS) Production by Congo Red

The slime-producing ability of *S. mutans* in the absence or presence of CBG was determined by using the Congo Red Agar method [41]. Three drops of 10 µL of an overnight culture of *S. mutans* were seeded on Congo Red agar plates containing various concentrations of CBG followed by a 24 h incubation at 37 °C in 95% air/5% CO_2_. The black color that appeared around the inoculum represents the amount of EPS produced. Image J program (The National Institute of Health, Bethesda, MD, USA) was used to analyze the area of the black color. The area of EPS was calculated by subtracting the area of the bacterial colony from the total black area. The Congo Red Agar plates were prepared by diluting 10 mL of an autoclaved 0.8% stock solution of Congo red stain in 100 mL of autoclaved BHI containing 1.5% agar to which glucose was added to a final concentration of 2%, and CBG added to the indicated final concentrations when the temperature had cooled down to 55 °C.

### 2.12. ROS Production

Untreated and CBG-treated *S. mutans* biofilms formed in 96-well black microtiter plates grown for 24 h were washed twice with PBS and exposed to 10 µM of 20,70-dichlorodihydrofluorescein diacetate (DCFH-DA) (Sigma-Aldrich, St. Louis, MO, USA) for 1 h at 37 °C [42]. DCFH-DA passively diffuses through the cell membrane into the cell, where it is deacetylated by esterases to form the non-fluorescent 2,7-dichlorodihydrofluorescein (DCFH). DCFH reacts with ROS to form the fluorescent product 2,7-dichlorofluorescein (DCF) [43]. After staining, the biofilms were washed with PBS, and the fluorescence intensities (FIs) of the biofilms were measured in an Infinite M200 PRO plate reader (Tecan) (excitation, 485 nm, emission, 535 nm). The FI values were normalized to the amount of metabolically active bacteria in parallel biofilms assessed by the MTT assay described above. The data are presented as a percentage of the control bacteria that have received equal concentrations of ethanol.

### 2.13. Metabolic Activity/Biomass Index

Biofilm of *S. mutans* were prepared as described above for 24 h at 37 °C. Then, the formed biofilms were washed twice with PBS and treated with various concentrations of CBG (4–20 μg/mL) or respective ethanol concentrations in BHI with 2% sucrose for 2, 4, 12, and 24 h. For each time point, the biofilms were washed twice with PBS, and parallel Crystal violet (CV) staining and MTT assay were performed. The metabolic activity/biomass index was calculated by dividing the MTT reading from each sample of each time point to the corresponding CV staining of the same sample.

### 2.14. Statistical Analysis

The experiments were performed independently three times in triplicates, and the data were analyzed statistically using Student’s t test in Microsoft Excel, with a *p* value of less than 0.05 considered significant when comparing treated versus control samples.

## 3. Results

### 3.1. CBG Reduces Biofilm Formation by S. mutans

**Figure 1:**
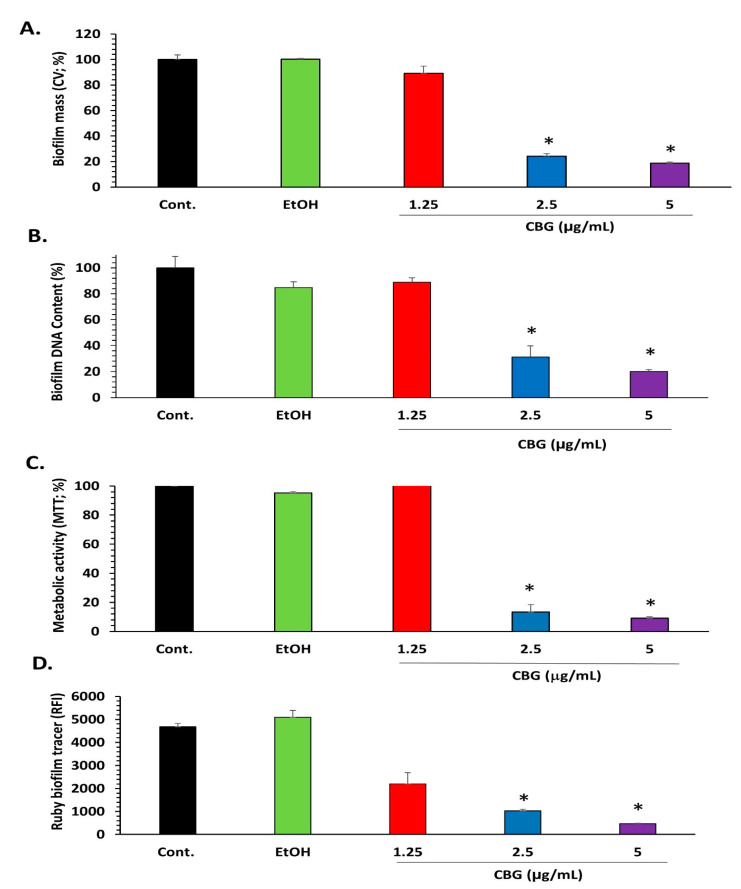
Anti-biofilm activity of CBG on *S. mutans*. (**A**) Biofilm mass of *S. mutans* that have been incubated in the absence or presence of various CBG concentrations or respective ethanol concentrations for 24 h as determined by crystal violet staining. *n* = 3; * *p* < 0.05. (**B**) Biofilm mass of untreated and CBG-treated *S. mutans* as determined by DNA content. *n* = 3; * *p* < 0.05. (**C**) Metabolic activity as measured by MTT reduction of the biofilm from untreated and CBG-treated *S. mutans*. *n* = 3; * *p* < 0.05. (**D**) FilmTracer SYPRO Ruby biofilm matrix staining of *S. mutans* biofilms formed for 24 h in the absence or presence of various concentrations of CBG. *n* = 3. * *p* < 0.05 in comparison with untreated bacteria.

**Figure 2:**
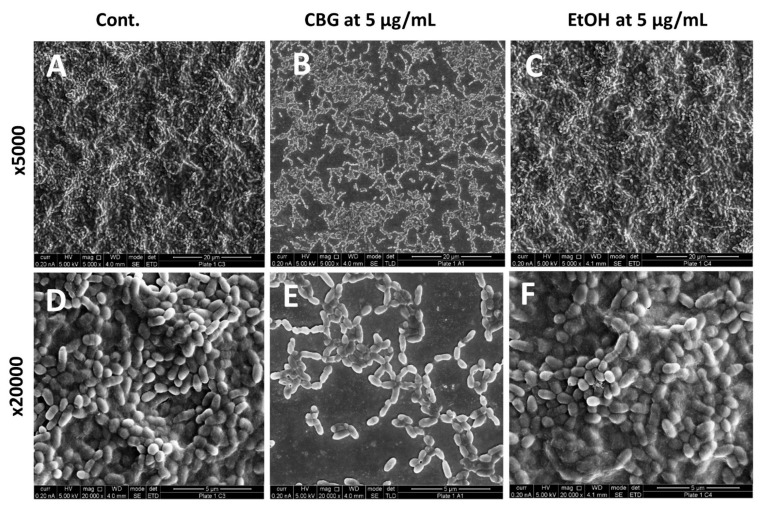
CBG reduces biofilm formation and prevents EPS production. HR-SEM images of untreated (**A**,**D**), CBG (5 µg/mL)-treated (**B**,**E**) and EtOH (0.05%)-treated (**C**,**F**) *S. mutans* (×5000 + ×20000 magnification). All treatments were applied for 24 h. The images are representative of three independent experiments.

We tested the effect of CBG on the biofilm formation of *S. mutans*. Crystal violet staining shows that CBG at concentrations equal to and higher than 2.5 µg/mL reduced the biofilm mass of *S. mutans* by more than 75% (Figure 1A; *p* < 0.05). Additionally, the DNA content and the metabolic activity of the biofilms were strongly reduced at concentrations equal to and higher than 2.5 µg/mL CBG (85–90% reduction) (Figure 1B,C; *p* < 0.05). When staining the biofilms with the SYPRO Ruby FilmTracer, a reduction in biofilm staining was also observed with 1.25 µg/mL CBG, although less pronounced than with 2.5 and 5 µg/mL CBG (Figure 1D; *p* < 0.05). The strong anti-biofilm activity of CBG on *S. mutans* was confirmed by HR-SEM (Figure 2). HR-SEM shows that CBG treatment results in single level bacterial culture with many empty interspaces and no visible EPS (Figure 2B,E), which is in stark contrast to the untreated and ethanol-treated control samples, where multiple complex levels of bacteria are seen embedded in an EPS matrix (Figure 2A,C,D,F). The HR-SEM shows that there are many hill-like and valley-like structures in the biofilms of the control samples (Figure 2A,C,D,F), which is not observed in the CBG-treated samples (Figure 2B,E).

### 3.2. CBG Altered the Roughness Profile Resulting in a Smoother Surface of S. mutans Biofilms

**Figure 3:**
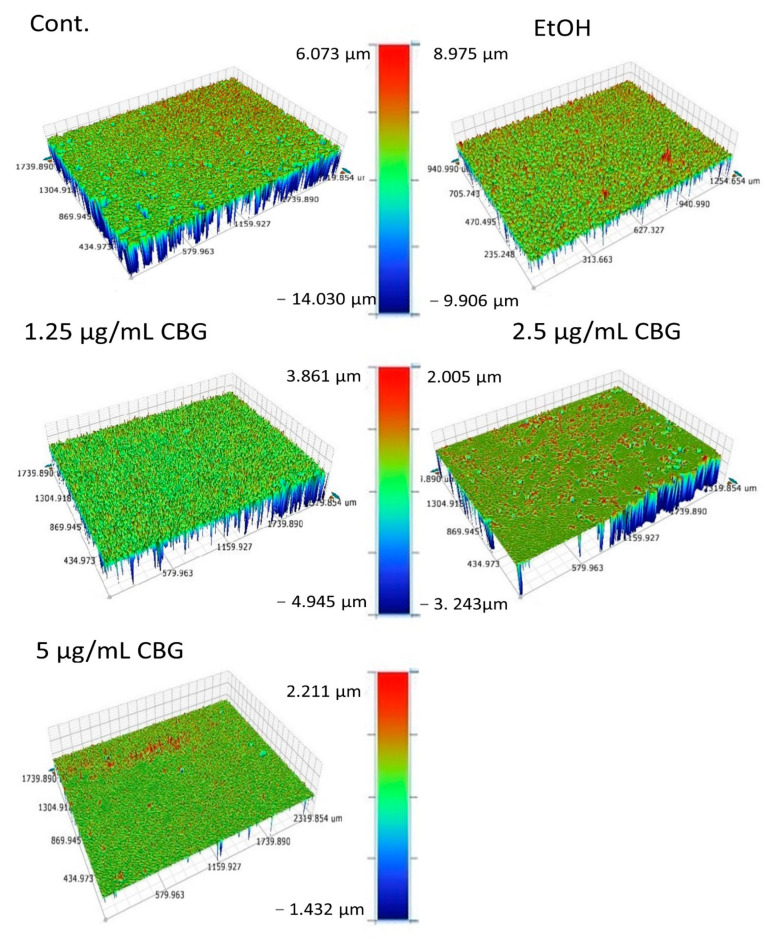
CBG causes a smoother *S. mutans* biofilm. Surface topography of untreated and CBG-treated *S. mutans* biofilm. Color scale denotes height and depth. The results are representative of three experiments.

Since HR-SEM is limited in observing the biofilm structure from above, we analyzed the effect of CBG on the topographical properties of *S. mutans* biofilms using an optical profilometer (OP). The OP allowed us to measure the profile roughness parameter (Ra), the maximum height (Rt), and maximum valley depth (Rv) of the control and the treated samples. Significant differences were found in the topography of the biofilms after exposure to CBG compared with the control samples (Figure 3). OP topographic images showed a rough surface with many valleys in the control biofilm (Figure 3) with an average Ra value of 998.27 ± 63 nm, Rt value of 19.59 ± 3.2 µm, and a Rv value of −11.24 ± 4.3 µm. Upon increasing concentrations of CBG (1.25, 2.5 and 5 μg/mL), the *S. mutans* biofilms become gradually smoother and thinner with smaller valleys (Table 2). At 5 µg/mL CBG, the Ra, Rt, and Rv values were 77.17 ± 13 nm, 2.93 ± 0.7 µm, and 1.66 ± 0.55 µm, respectively. These data demonstrate that CBG alters the biofilm structure in addition to reducing the biomass.

### 3.3. CBG Decreases EPS Production by S. mutans

**Figure 4:**
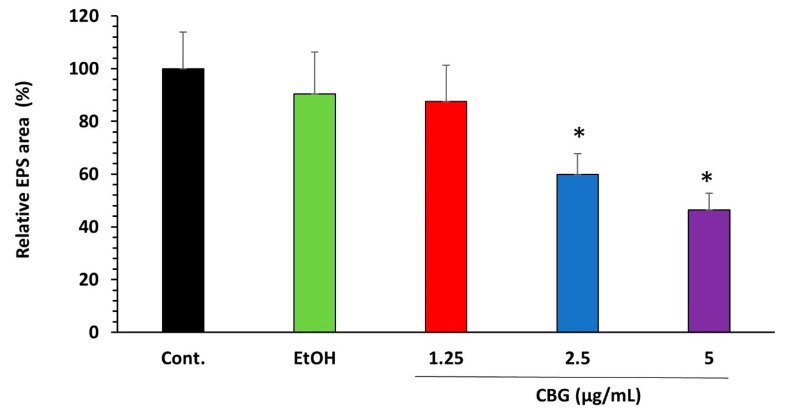
CBG decreases EPS production in *S. mutans* biofilm. Congo red matrix staining of *S. mutans* biofilms formed for 24 h in the presence of various concentrations of CBG introduced into the agar plates. *n* = 3. * *p* < 0.05.

*S. mutans* is a key contributor to the formation of the EPS matrix in dental biofilms [19]. Therefore, it was important to analyze the effect of CBG on EPS production by *S. mutans*. The EPS production was studied by seeding the bacteria on Congo red agar plates containing different concentrations of CBG in the presence of 2% sucrose. Although black colonies were observed on both control and CBG-containing agar plates, indicating that EPS is still produced, the amount of EPS formed around the bacterial colonies was significantly reduced in the presence of CBG when compared with the control (Figure 4; *p* < 0.05).

### 3.4. CBG Increased the Intracellular Reactive Oxygen Species (ROS) Levels

**Figure 5:**
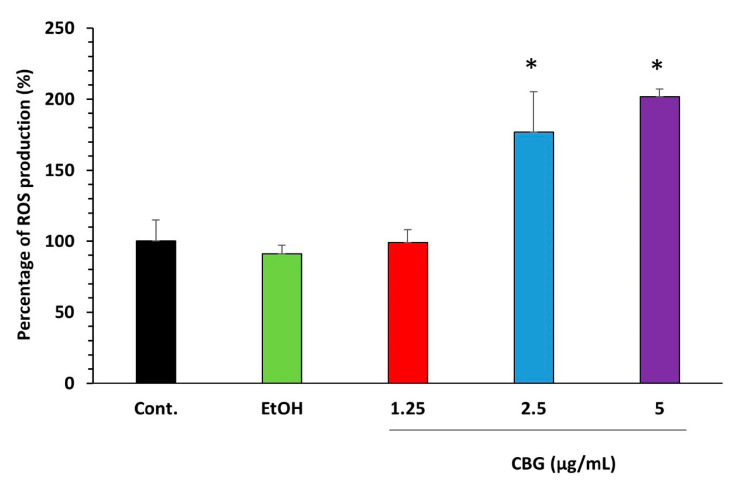
Effect of CBG on ROS accumulation. *S. mutans* biofilms formed in the absence or presence of CBG were loaded with DCFH-DA and the relative fluorescence intensities (RFI) of the biofilms were measured in a plate reader. The RFI values were normalized to the amount of metabolically active cells in biofilms assessed by MTT assay. *n* = 3. * *p* < 0.05.

Oxidative stress generated by reactive oxygen species has a major impact on the establishment, persistence, and virulence of *S. mutans* [44]. We therefore studied the effect of ROS production in CBG-treated biofilms. Biofilms that have been stained with the ROS indicator DCFH-DA demonstrated an increase in green fluorescence intensity upon treatment with 2.5 µg/mL and 5 µg/mL CBG in comparison with the untreated and ethanol-treated control samples (Figure 5; *p* < 0.05), indicating that CBG induces intracellular ROS production in *S. mutans* biofilms.

### 3.5. CBG Reduces the Expression of Various Genes Involved in Essential Metabolic Pathways Related to the Cariogenic Properties of S. mutans

**Figure 6:**
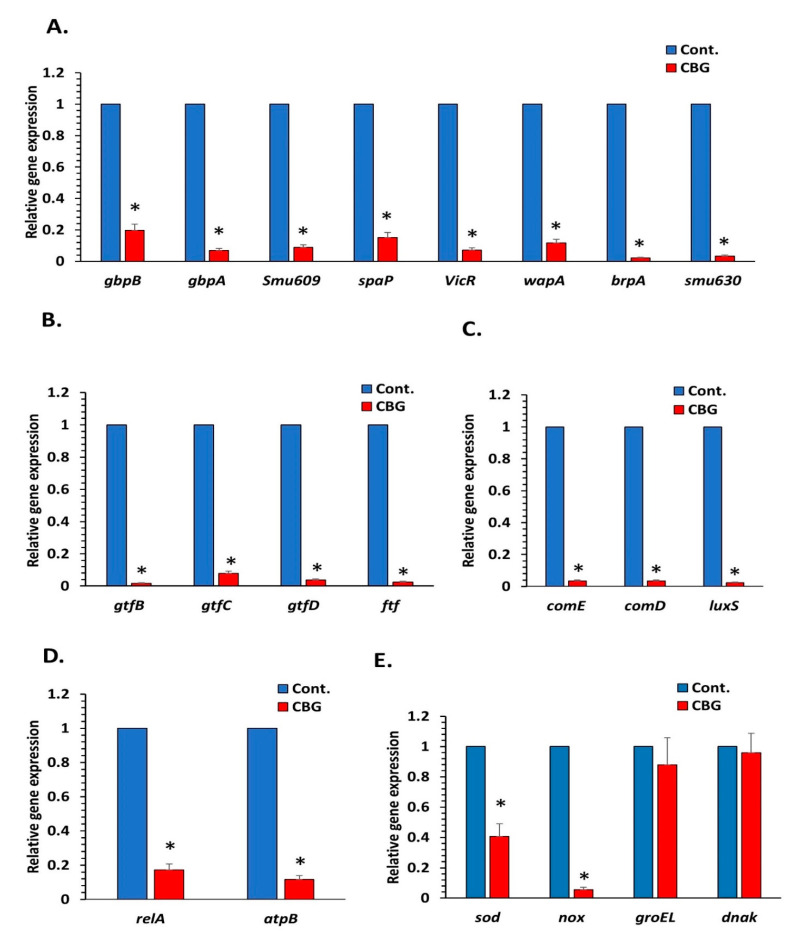
Effect of CBG on gene expression in *S. mutans* biofilm. Gene expression in CBG-treated *S. mutans* biofilm (2.5 µg/mL, 24 h) in comparison with untreated control as quantified by real-time PCR using respective primers. (**A**). Biofilm formation-related genes. (**B**). EPS formation-related genes. (**C**). Quorum sensing-related genes (**D**). Acid tolerance-related genes. (**E**). Antioxidant and stress responsive-related genes. *n* = 3. * *p* < 0.05.

The cariogenic properties of *S. mutans* biofilms are regulated by various genes, which include the five essential metabolic pathways: microbial adhesion, biofilm formation, extracellular polysaccharide synthesis, quorum sensing, and acid tolerance. It was querying to analyze changes in the expression of genes involved in these processes in *S. mutans* biofilms after treatment with CBG. We decided to use 2.5 µg/mL CBG, which is the minimum biofilm inhibitory concentration (MBIC) for *S. mutans* (Figure 1). Genes involved in microbial adhesion (1a-sucrose-dependent adhesion: *gbpB*, *gbpA*, and *wapA*; 1b-sucrose-independent adhesion: spaP) and biofilm formation (*brp*, *smu630*, *smu609*, *vicR*, and *wapA*) are significantly downregulated in biofilms formed in the presence of 2.5 µg/mL CBG in comparison with control samples when using 16S rRNA as the internal standard (Figure 6A; 80–98% reduction with a *p* < 0.05). Additionally, genes involved in extracellular polysaccharide synthesis (*gtfB*, *gtfC*, *gtfD*, and *ftf)* (Figure 6B; *p* < 0.05), quorum sensing (*comE*, *comD*, and *luxS*) (Figure 6C; *p* < 0.05), and acid tolerance (*atpB* and *relA*) (Figure 6D; *p* < 0.05) were strongly downregulated (80–98%). Notably, both sodA and nox involved in oxidative stress defense were downregulated by CBG (Figure 6E), which could explain the increase in ROS level. In contrast, the stress-associated genes *groEL* and *dnaK*, which are induced by heat shock and acidic stress [45,46], were not significantly affected by CBG.

### 3.6. CBG Decreases the Metabolic Activity of Preformed Biofilms

**Figure 7:**
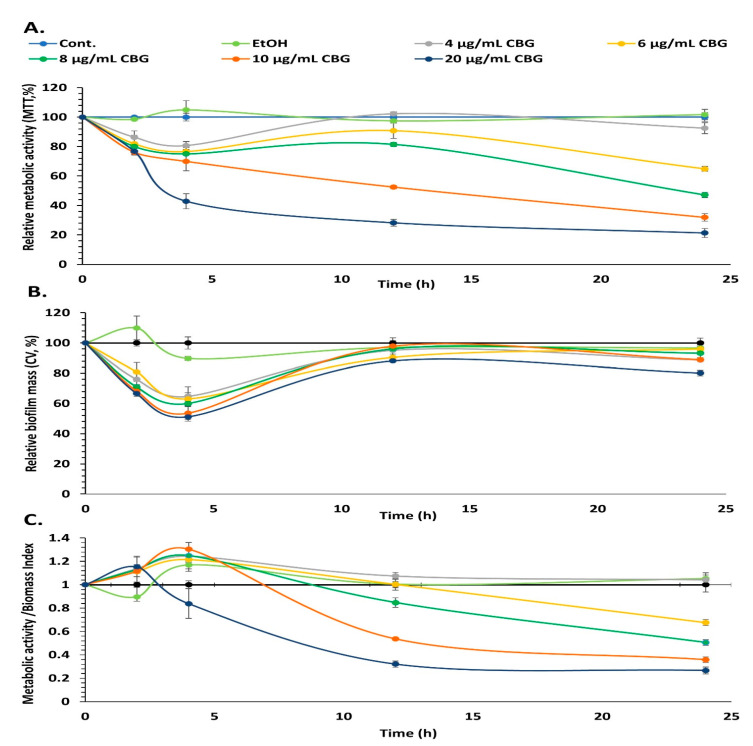
CBG decreases the metabolic activity of preformed biofilms. *S. mutans* was allowed to form biofilms for 24 h prior to exposure to various concentrations of CBG, and the biofilm metabolic activity (**A**) and biofilm mass (**B**) were measured at various time points using the MTT assay and CV staining method, respectively. (**C**) The metabolic activity/biofilm mass index of samples presented in A and B. *n* = 3.

To investigate whether CBG could destroy preformed biofilms, biofilms were allowed to form for 24 h before treatment with various concentrations of CBG (0–20 µg/mL). The metabolic activity and biofilm biomass were assessed by CV staining and MTT assay at various time points (0–24 h) after adding CBG and compared with control samples. Both the metabolic activity (Figure 7A) and the biofilm mass (Figure 7B) were reduced by CBG after 2 and 4 h incubation. During the first 4 h of treatment, the reduction in biofilm biomass by CBG was more profound (Figure 7B; 40–50%) than the reduction in the metabolic activity (Figure 7A; 20–25%) at all tested CBG concentrations except for 20 µg/mL. After 12 h, the biomass of CBG-treated biofilms recovered and reached a similar level to the control bacteria (Figure 7B), while the metabolic activity was still suppressed (Figure 7A). The metabolic activity/biomass index was strongly reduced by 10 and 20 µg/mL CBG after 12 and 24 h incubation (26–35% of control) (Figure 7C). CBG at 6 and 8 µg/mL reduced this index after 24 h. Thus, CBG can reduce the metabolic activity of preformed biofilms.

## 4. Discussion

Many microorganisms in dental plaque have been found to be associated with dental caries; among them, *S. mutans* is considered the most cariogenic bacterium [47]. Therefore, the inhibition of *S. mutans* biofilm formation is a key target to forestall dental caries. The present study shows that the cannabinoid CBG exerts strong anti-biofilm activities towards *S. mutans* at a concentration as low as 2.5 µg/mL and, thus, is a potential drug in preventing dental caries. The same concentration was found to inhibit the planktonic growth of *S. mutans* [34], suggesting that some of the effects on biofilm formation is due to its direct anti-bacterial activity. This is demonstrated by the reduced metabolic activity observed after treating preformed biofilms with CBG. Previous data show that CBG induces an immediate membrane hyperpolarization and increases the membrane rigidity, which is followed by an increase in the membrane permeability [34]. These effects of CBG might contribute to the reduced metabolic activity of preformed biofilms. CBG also prevented the drop in pH caused by *S. mutans* that correlated with the reduced bacterial growth [34].

Our data also indicate that CBG has a direct anti-biofilm activity. Many of the genes involved in the various metabolic pathways involved in biofilm formation were downregulated by CBG. Among the genes suppressed by CBG are *gbpB* essential for *S. mutans* growth [48], *vicR* associated with cell wall biogenesis and biofilm formation [49], *brpA* regulating the development of mature *S. mutans* biofilms [50], and the wall-associated protein A (*wapA*) affecting sucrose-independent cell–cell aggregation and biofilm architecture [51]. Moreover, CBG reduces the expression of the cell surface antigen (*spaA*), which mediates the binding of *S. mutans* to tooth surfaces [52]. Taken together, the CBG-induced changes in gene expression would interfere with the appropriate development of biofilms, which may explain the anti-biofilm activity of this compound.

Another important finding is that CBG reduces EPS production. This activity can, in part, be explained by the reduced expression of genes regulating EPS production including *gtfB, gtfC gtfD*, and *ftf*. Since EPS functions as a protective barrier against various stress stimuli and prevents the penetration of several antibiotics [53], the inhibition of EPS production by CBG is important to enable the action of other anti-bacterial compounds.

CBG treatment led to increased ROS levels that might cause oxidative stress in the bacteria. This observation is in accordance with the data observed by Feldman et al. [54], demonstrating an increase in ROS production in *C. albicans* biofilm treated with CBD. Additionally, Singer et al. [55] observed that CBD induces robust ROS production when using glioma stem cells. Usually, bacteria contain protective proteins that can detoxify reactive oxygen species (ROS), but if the ROS production becomes uncontrolled, it might be cytotoxic to the bacteria [56]. Therefore, we studied the expression of the O_2_-consuming NADH oxidase (*nox*) and the antioxidant enzyme sodium dismutase (*sodA*) that are involved in the protection against oxidative stress. Interestingly, CBG caused a strong downregulation of both genes (90% reduction of *nox* and 60% reduction of *sodA*). Thus, CBG treatment makes the bacteria more susceptible to oxidative stress stimuli. On the other hand, the two stress response genes, *groEL* and *dnaK*, were not significantly changed by CBG. These proteins are usually induced by heat shock and acidic stress stimuli [45,46].

Bacterial biofilm formation is associated with quorum sensing [33]. CBG caused a marked reduction in the gene expression of two quorum sensing-associated pathways, namely *luxS*, which synthetizes autoinducer 2 (AI-2), and *comD* and *ComE* involved in sensing and response regulation of competence stimulating peptide (CSP). We have previously observed that CBG prevents quorum sensing in the Gram-negative *Vibrio harveyi* by increasing LuxO expression and activity, with a concomitant downregulation of the *LuxR* gene [33]. Therefore, CBG might also act as an anti-quorum sensing agent.

Importantly, CBG reduced the metabolic activity in pre-formed *S. mutans* biofilms, which might be due to its anti-bacterial activity. The fact that CBG might affect the viability of *S. mutans* embedded in a biofilm means that this drug can penetrate the biofilm and that the sessile biofilm-associated bacteria are still sensitive to the drug. Thus, the action of CBG differs from many antibiotics that require active dividing bacteria. The susceptibility of antibiotic resistance *S. aureus* [29] to the anti-bacterial effect of CBG suggests that the drug-resistant mechanisms in these bacteria do not interfere with the anti-bacterial activity of CBG.

CBG is currently marketed as a dietary supplement and, similar to cannabidiol (CBD), many claims have been made about its benefits. Thus far, CBG has been shown to exert diverse effects in eukaryotes [33], but much less is known about its actions on bacteria. Although there are some studies that have shown an anti-bacterial activity of CBG towards Gram-positive bacteria [29,34,57], still, further investigations are warranted to identify potential areas of therapeutic uses and hazards. Here, we propose CBG as a novel agent to combat dental diseases such as dental caries. It is also expected to be beneficial as an active ingredient against periodontal diseases. The anti-bacterial/anti-biofilm activities would protect from the bacterial load, while its anti-inflammatory properties [28] would aid in gum repair.

The present research was performed in vitro to explore the potential effects of CBG on *S. mutans*—the main causative agent of dental caries. Since dental caries is also caused by a variety of other microorganisms, further studies should be focused on the ability of CBG to act in heterogenous cultures as well as using models that mimic the oral environment.

## 5. Conclusions

In conclusion, CBG inhibits the formation of biofilms indirectly by acting as an anti-bacterial agent and directly by acting on metabolic pathways regulating biofilms. It reduces the expression of essential biofilm-regulating genes, prevents EPS production, inhibits quorum sensing, and increases ROS production. In addition, it suppresses the metabolic activity of the bacteria. Its dual anti-bacterial/anti-biofilm activities on *S. mutans* make it a potential drug in the prevention of tooth decay.

## Figures and Tables

**Table 1:** Primers used for real-time PCR.

Gene	Forward Primer	Reverse Primer
*16S rRNA*	CCTACGGGAGGCAGCAGTAG	CAACAGAGCTTTACGATCCGAAA
*atpB*	AGCCAACCTTGGCAACTGAAA	TGTCAGACGGCGTTCAAGGTT
*brpA*	GGAGGAGCTGCATCAGGATTC	AACTCCAGCACATCCAGCAAG
*comD*	TGAAAATAGCATAGGTGAGTCAAAG	ATTTAGGTTAGCTGATTAACACTATACAC
*comE*	CACAACAACTTATTGACGCTATCCC	TGATTGGCTACTTCCAGTCCTTTC
*dnak*	GCAGGTCAAGAGGGAGCTCA	CCGCCCTTGTCTGAGAATC
*gbpA*	GGTGGTTCTGTGCCTGATGA	TTGCCAGCCTGATACACGTT
*gbpB*	AGGGCAATGTACTTGGGGTG	TTTGGCCACCTTGAACACCT
*groEL*	CCAGGAGCTTTGACTGCGAC	TTGCGGATGATGATGTAGATGGT
*gtfB*	AGCAATGCAGCCAATCTACAAAT	ACGAACTTTGCCGTTATTGTCA
*gtfC*	GGTTTAACGTCAAAATTAGCTGTATT	CTCAACCAACCGCCACTGTT
*gtfD*	CAGGCAGCCAACGCATTAA	AGCCCTCGCTCATCATAAGC
*luxS*	ACTGTTCCCCTTTTGGCTGTC	AACTTGCTTTGATGACTGTGGC
*nox*	GGGTTGTGGAATGGCACTTTGG	CAATGGCTGTCACTGGCGATTC
*relA*	ACAAAAAGGGTATCGTCCGTACAT	AATCACGCTTGGTATTGCTAATTG
*soda*	GGCTCAGGTTGGGCTTGGTTAG	GCGTGTTCCCAGACATCAAGTGC
*spaP*	GACTTTGGTAATGGTTATGCATCAA	TTGCCAGCCTGATACACGTT
*vicR*	CGCAGTGGCTGAGGAAAATG	ACCTGTGTGTGTCGCTAAGTGATG
*wapA*	GCACGCTTGCAGTACATTGC	CATAAGGTCGCGAGCAGCT

**Table 2:** Table of roughness profile: roughness parameter (Ra), the maximum height (Rt), and maximum valley depth (Rv). The results are representative of three experiments. N = 3. *p* < 0.05.

Title 1	Control	EtOH (0.05%)	1.25 μg/mL CBG	2.5 μg/mL CBG	5 μg/mL CBG
Ra (nm)	998.27 ± 63	988.20 ± 82	270.54 ± 59	100.32 ± 13	77.17 ± 13
Rt (µm)	19.59 ± 3.2	21.97 ± 5.9	6.66 ± 1.2	4.96 ± 2.7	2.93 ± 0.7
Rv (µm)	−11.21 ± 4.3	−12.22 ± 2.7	−3.03 ± 1.8	−2.35 ± 1.9	−1.66 ± 0.55

## Data Availability

Raw data for the figures are available upon reasonable request from the corresponding author.
